# Upcycling of Poly(Lactic Acid) by Reactive Extrusion with Recycled Polycarbonate: Morphological and Mechanical Properties of Blends

**DOI:** 10.3390/polym14235058

**Published:** 2022-11-22

**Authors:** Vito Gigante, Laura Aliotta, Maria-Beatrice Coltelli, Andrea Lazzeri

**Affiliations:** 1 Department of Civil and Industrial Engineering, University of Pisa, 56122 Pisa, Italy; 2 National Interuniversity Consortium of Materials Science and Technology (INSTM), 50121 Florence, Italy

**Keywords:** reactive extrusion, mechanical behavior, recycled polymers, blending

## Abstract

Poly(lactic acid) (PLA) is one of the most promising renewable polymers to be employed to foster ecological and renewable materials in many fields of application. To develop high-performance products, however, the thermal resistance and the impact properties should be improved. At the same time, it is also necessary to consider the end of life through the exploration of property assessment, following reprocessing. In this context the aim of the paper is to develop PLA/PC blends, obtained from recycled materials, in particular scraps from secondary processing, to close the recycling loop. Indeed, the blending of PLA with polycarbonate (PC) was demonstrated to be a successful strategy to improve thermomechanical properties that happens after several work cycles. The correlation between the compositions and properties was then investigated by considering the morphology of the blends; in addition, the reactive extrusions resulting in the formation of a PLA-PC co-polymer were investigated. The materials obtained are then examined by means of a dynamic-mechanical analysis (DMTA) to study the relaxations and transitions.

## 1. Introduction

Recycling plastics is one of the best opportunities available to reduce pollution, save raw materials, store carbon and protect ourselves from the negative effects of waste dispersion in nature and in the sea [1]. This assumption is also confirmed by the European Community directives that, starting from 2015, adopted an action plan aimed at fostering Europe’s transition into a circular economy, in which waste is not simply disposed of but transformed into a valuable secondary raw material for further production [2]. There are a lot of reasons to incentivize and encourage plastic recycling, including limiting the use of landfills, optimising resources, limiting CO_2_ emissions related to plastic production processes into the atmosphere and nurturing virtuous supply chains that can create sustainable employment [3].

In this framework, it is of great interest, both scientifically and in terms of industrial upscaling, to succeed in designing ‘circular-by-design’ materials, i.e., materials that can have such characteristics as to be recyclable after use, especially for high value-added applications. In addition, a topical challenge is to introduce in the recycling lines, renewable polymers, making them attractive for even high-performance applications through modifications and blending with other polymers. In this respect, the feasibility of a large window of production processes has guaranteed that poly(lactic acid) (PLA) is attractive for this purpose [4]. However, PLA is an extremely brittle material, it has a low toughness, it shows a low thermal resistance and it is relatively hydrophobic (with a static contact angle with water of 80°) [5]. To compensate for these defects (and above all to increase the impact properties) the incorporation of additives, such as inorganic particles or blending with tough polymers can be found in the literature [6,7,8]. Indeed, blending PLA with engineering plastics, such as polycarbonate (PC), because of its high Tg, high thermal stability, high tensile strength, and elongation at break, is a successful strategy to improve PLA properties, as confirmed by several studies [9,10,11,12,13]. The processing of PLA/PC blends can occur at temperatures lower than those typical of pure PC processing and this represents an opportunity to recycle post-consumer PC with a reduced energy consumption. Nevertheless, it has been demonstrated that PLA/PC blends are immiscible, so the adhesion between the two polymers is weak due to the high surface tension; over time, different methods of compatibility have been investigated: through the use of poly(butylene succinate-co-lactate) (PBSLA) [14] or poly(ethylene-co-vinyl) EVA [15] to form ternary systems. Ikehara et al. [16] used a biodegradable semi-crystalline polycarbonate called PEC (polyester carbonate) to verify that spherulites of the two polymers can interpenetrate, Wang et al. blended PC and PLA with an epoxidizing catalyst, which improved the toughness through a better compatibility [17].

The study of the effect of various catalysts (zinc borate, titanium, tetrabutyltitanate pigments) [18,19] on the interchange reactions of PLA and PC was investigated. The idea was to follow the path of PC/PBT [20] or PC/PET [21] blends where copolyesters were formed by a transesterification reaction and improved the compatibility of the two polymers.

The increase in the interfacial adhesion between PLA and PC by using catalysts that favor interchange reactions (capable of providing a new type of copolymer with high mechanical properties and an excellent thermal resistance), is the approach also used by Phuong et al. [22,23]. but with the step ahead to minimize the reaction times, making this solution exploitable, even in industrial processes where residence times in the extruder are limited.

As described above, the recycling of waste and discarded plastics is desirable for environmental and economic reasons. However, recycling of the PLA/PC blends is still in its early stages. Post-consumer recycling of the PLA/PC blends has been simulated in a few works [24,25], and the results clearly showed that aging corresponding to one year of use leads to the significant degradation of PLA, resulting in a reduced elongation at break. In addition, the content of PLA as a biopolymer in the PLA/PC blends should be as high as possible once performance requirements are met, which is helpful in reducing the environmental impact [26].

For all these reasons, the purpose of the present work is to implement knowledge on the recyclability of these blends, starting with a comparison of the process and mechanical properties of the PLA and PC scraps recycled from thermoforming and virgin polymers. In addition, it was sought to understand whether a catalytic system consisting of Triacetin (TA) and TetraButylAmmonium TetraPhenylBorate (TBATPB) patented by some of the authors of this paper [27] could also act as a compatibilizer for the recycled PLA (R-PLA)/recycled PC (R-PC) blends, by adding the catalyst during the extrusion to analyze the possibility of the improved adhesion between the components. The presence of the PLA-PC copolymers can be detected by means of a thermal-dynamic-mechanical analysis (DMTA) [28]: a new glass transition temperature intermediate between those of pure PLA and PC will demonstrate the formation of a new species.

With the intention of including as much PLA as possible, but at the same time achieving thermomechanical properties comparable with petro-based blends for durable applications, in this work, the recycled PLA/PC co-continuous blends were developed and produced [29,30], i.e., to compare the PLA matrix blends with the PC dispersed phase, and vice versa, while also investigating the differences in the presence and absence of the catalytic system. Through this study, therefore, the aim is to combine the need to increase knowledge of the thermomechanical properties of recycled polymeric materials, including renewable polyesters, and the opportunity to improve their interfacial adhesion.

## 2. Materials and Methods

### 2.1. Materials

PLA: Poly(lactic acid) 2003D (Natureworks LLC, Minnetonka, MN, USA) is a thermoplastic resin, derived from renewable resources (corn starch or sugar cane), transparent, with a high PM (around 200,000 g/mol) density of 1.24 g/cm^3^, processable by extrusion, injection molding and thermoforming. It has an amount of around 4 percent of D units that introduce imperfections in the helical conformation of the polymer and defects in the crystal arrangement. It is routinely used as part of polymer blends.

PC: Bisphenol A Polycarbonate S3000 (Mitsubishi Chemical Co., Tokyo, Japan) with a density of 1.20 g/cm^3^ and a molecular weight of 56,000 g/mol.

R-PLA: In this paper, reprocessed PLA2003D will be defined with the name of R-PLA. The regrinding of the PLA scraps has been carried out by Romei s.r.l (Florence, Italy).

R-PC: With the label R-PC, the post-industrial Bisphenol A Polycarbonate S3000, consisting of scraps of grey color, has been defined. The regrinding of the PC scraps has been accomplished by Romei s.r.l (Florence, Italy).

CATA: Triacetin (TA) and TetraButylAmmonium TetraPhenylBorate (TBATPB), both purchased from Sigma-Aldrich (Merk Life Science S.r.l., Milano, Italy) were used as catalysts for the interchange reactions, following the process described in [22].

Firstly, virgin PLA and virgin PC were compared with their recycled counterparts; thereafter the blends with a co-continuous morphology were produced and characterised, to evaluate whether the catalytic system acts as compatibilizer (Table 1).

### 2.2. Processing

A conic twin-screw micro compounder (ThermoScientific HAAKE MiniLab II, Karlsruhe, Germany) was used to process and extrude the polymeric blends with and without the catalytic system. The melt filament was collected by a heated cylinder piston and fed into a mini-injection molding machine (Thermo Scientific HAAKE Minijet II, Karlsruhe, Germany), to produce specimens for the tensile tests (25 × 5 × 1 mm) and for the impact/fracture properties (80 × 10 × 4 mm). The processing temperature selected for the blends with and without CATA was 235 °C, the mold was held at 60 °C for an injection cycle of 25 s. Regarding PLA and R-PLA, the extrusion temperature was set at 190 °C, whereas for PC and R-PC, it was set at 280 °C.

### 2.3. Testing Methodologies

Firstly, during the micro compounding process, the torque trend over time, was closely related to the viscosity of the fluid itself, and was evaluated to understand the variations in the melt strength among the various formulations tested.

The quasi-static tensile tests were carried out at room temperature on Haake type III dog-bone tensile bars (size: 25 × 5 × 1.5 mm), at a crosshead speed of 10 mm/min by an Instron 5500R universal testing machine (Canton, MA, USA), equipped with a 1 kN load cell and interfaced with a computer, running MERLIN software (INSTRON version 4.42 S/N–14733H).

The impact tests were performed on V-notched 80 × 10 × 4 mm specimens, using a 15 J Instron CEAST 9050 Charpy pendulum (INSTRON, Canton, MA, USA) following the standard procedure ISO 179.

The dynamic mechanical thermal analysis (DMTA) was performed on a on a Gabo Eplexor^®^ 100N (Gabo Qualimeter GmbH, Ahlden, Germany). The test bars were of a size of 10 × 5 × 1.5 mm and placed on a tensile geometry configuration. The temperature used in the experiment ranged from −100 °C to 200 °C with a heating rate of 2 °C/min and a frequency of 1 Hz. The properties measured under this oscillating loading are the storage modulus (E′) and tan δ. The E′ value represents the stiffness of a viscoelastic material and is proportional to the energy stored during a loading cycle; tan delta is the ratio between the loss and storage modulus.

The morphology of the composites was studied by scanning electron microscopy (SEM) using JSM-5600LV (JEOL, Tokyo, Japan) and by analyzing the fractured surfaces of the samples obtained by breaking them in liquid nitrogen. Prior to the SEM analysis, all of the surfaces were sputtered with gold.

## 3. Results

Firstly, a preliminary study was performed, aimed at understanding whether PLA and PC recycled (R-PLA and R-PC) from industrial scraps kept rheological, processed and mechanical properties similar to virgin polymers. For this purpose, the measurements of the torque during the mixing time, the quasi-static tensile tests and the impact tests were carried out and discussed.

### 3.1. Comparison PLA/R-PLA and PC/R-PC

#### 3.1.1. Torque Analysis

**Figure 1:**
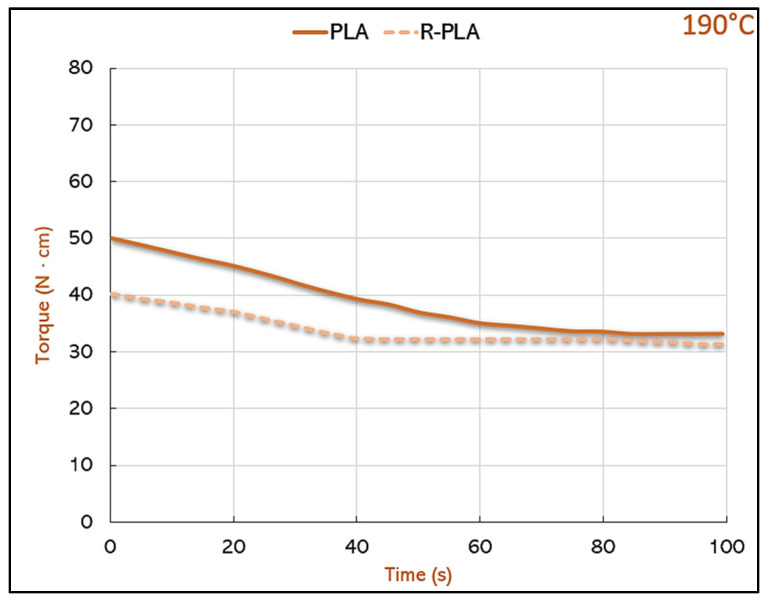
Torque trend for PLA and R-PLA.

It is accurate to assert that the viscous torque is a measure of the resistance that a fluid offers to the rotational motion of the conic twin screws and it is a function of the viscosity of the fluid itself [31]. By completely filling the micro compounder chamber (6 g), it can be observed that the higher the torque value, the higher the viscosity of the polymer. In Figure 1, the torque/time curves were recorded at 190 °C for PLA and R-PLA, over the extrusion time; the trend observed showed that for PLA, at time zero, the torque value turned out to be slightly higher than that of R-PLA, thus demonstrating the liability of the shorter chains after cleavages induced by the compounding, thermoforming and the second extrusion process suffered by R-PLA. Nevertheless, the difference tapers off as the mixing process advances, reaching similar torque values for PLA and R-PLA after 60 s. While the virgin PLA meets a continuous decrease in torque, associated with the chains breakage that is occurring for the first time as it not was not processed previously. R-PLA, instead, displayed a non-constant decrease, indeed after 40 s there is the presence of a plateau and, thus, the stabilization of the process conditions. The appropriate extrusion time to obtain the molten material to be transferred into the injection press lasted 60 s, however, we wanted to push the mixing time up to 100 s, in order to understand the torque trend, confirming that the torque of R-PLA remained stable up to 100 s, thus it was not going to encounter further cleaving or degradation; on the contrary, PLA continued to decrease its torque value for almost the entire test time.

**Figure 2:**
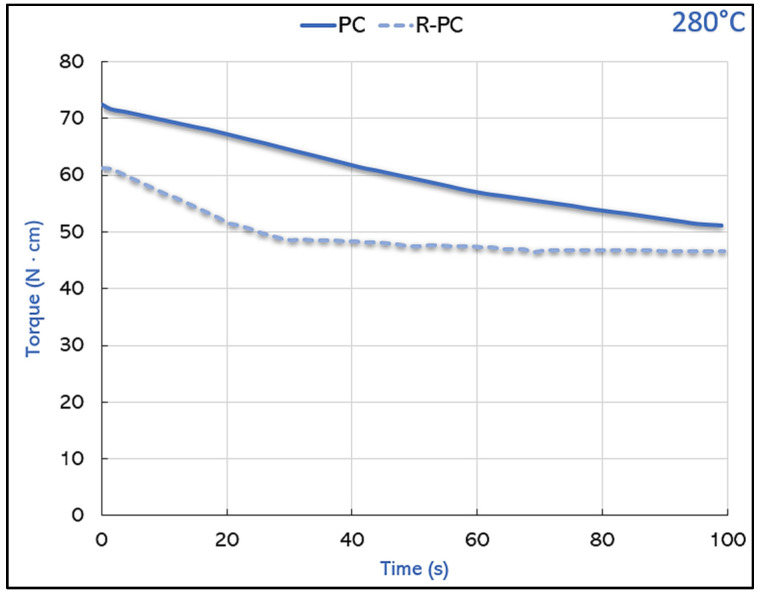
Torque trend for PC and R-PC.

The shape of the trend is mirrored in Figure 2 at 280 °C, regarding PC and R-PC. At their congenial extrusion temperature, they showed an almost continuous and constant decrease, concerning the virgin PC, basically matching what was reported by Chiu et al. [32], while the achievement of the torque stabilization was registered for R-PC, which started, as did R-PLA, from a relatively lower value than the virgin one, but it stabilized after 25 s and recorded a plateau. In the past, the decrease in the molecular weight of melt processed polycarbonate, was evidenced in different papers [33,34]. Similar papers were published about the PLA processing, proving the occurrence of a chain scission and the consequent decrease in the molecular weight [35,36]; indeed the rheological properties and the solution viscosity are very sensitive to the molecular weight changes and the correlations between the molecular weight and viscosity [37,38].

#### 3.1.2. Mechanical Properties

**Figure 3:**
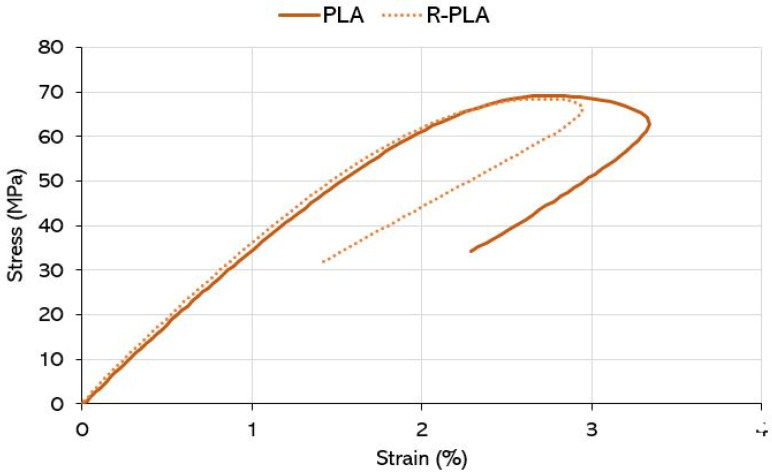
Stress/strain curves for PLA and R-PLA.

Starting from the PLA/R-PLA comparison, from the point of view of the tensile properties (two representative stress/strain graphs in Figure 3), except for the elongation at break, there are no substantial differences. These results can probably be ascribed to the struggle between some phenomena that occur contemporarily during the reprocessing, as the decrease of viscosity (registered by the torque decrement) is probably due to the molecular chain scission [39,40]. This is crucial feedback, because it allows for the reuse of the material for subsequent processing, as it has similar properties. Definitely, the PLA matrix blends are fragile and show a low elongation at break, whereas the bi-continuous blends, including a continuous PC phase, are better and show an improved elongation at break.

**Figure 4:**
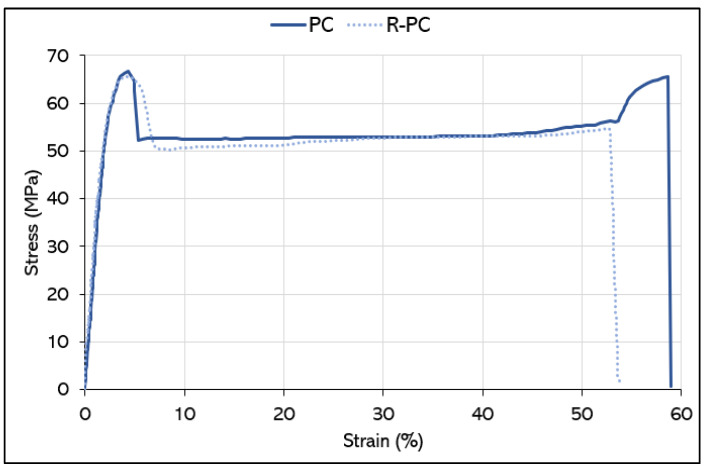
Stress/strain curves for PC and R-PC.

Furthermore, regarding the PC/R-PC comparison (where two representative curves are depicted in Figure 4), there is a slight lowering of the stress at break and elongation at break, again caused by reprocessing. A 3% degree decrease of the mechanical strength and stiffness of polycarbonate, after two reprocessing steps, is in line with what was found by Perez et al. [41] and more recently, by Reich et al. [42], in their studies on the mechanical property variations of polycarbonate after successive stages of extrusion/molding.

The results of the Charpy tests, shown in Table 2, reveal that the impact strength of the virgin PLA is slightly higher than the impact resistance of the recycled polymer; a reduction of around 5 percent in this property after the subsequent rework is what was also evaluated by Aguero et al. [43], who reprocessed PLA up to six times, at which point the lowering of the impact properties reached almost 50%. This progressive decrease in the energy absorption capacity can be linked with the degradation process on PLA. A 15% decrease has been evaluated for the impact resistance of R-PC, with respect to PC; but the value still turns out to be very useful for blending it with R-PLA and improving its impact resistance.

### 3.2. Blend Characterizations

#### 3.2.1. Torque Analysis

**Figure 5:**
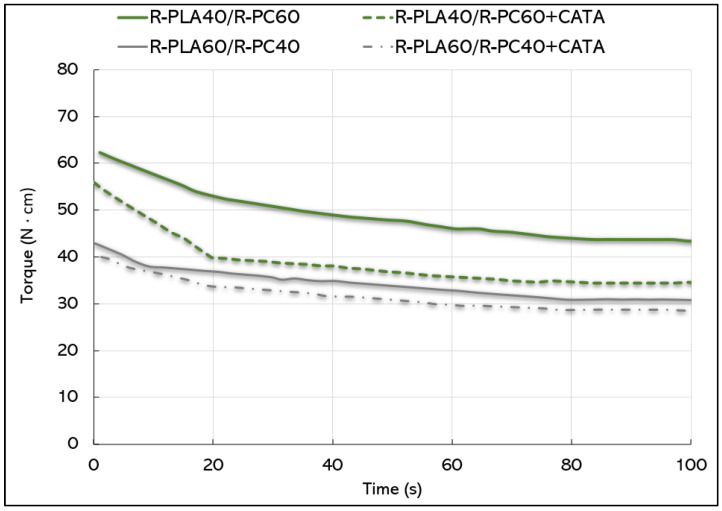
Torque trend for the R-PLA/R-PC blends.

Tests for the torque evaluation were performed on the studied blends at the same extrusion temperature (Figure 5). As for the pure polymers, a decrease in viscosity is shown for all blends with the increase of the dwell time in the micro compounder recirculation chamber. The trend decreased abruptly in the first 20 s, then a stabilization of the fluidity was registered.

The significant data concerning all of the materials, is that the addition of the catalytic system decreases the torque value with respect to the relative counterparts without TA and TBATPB; the reason is to be found in the typical process of the chain scission in the presence of a catalyst that polymers undergo during the transesterification reaction in the melt, with a decrease in the molecular weight and, consequently, of the torque value [44]. This difference is more pronounced for blends in which there is a higher amount of PC. These considerations should be related to the morphology of the blends that are analyzed in the following section.

#### 3.2.2. Morphological Structure and the Mechanical Results

**Figure 6:**
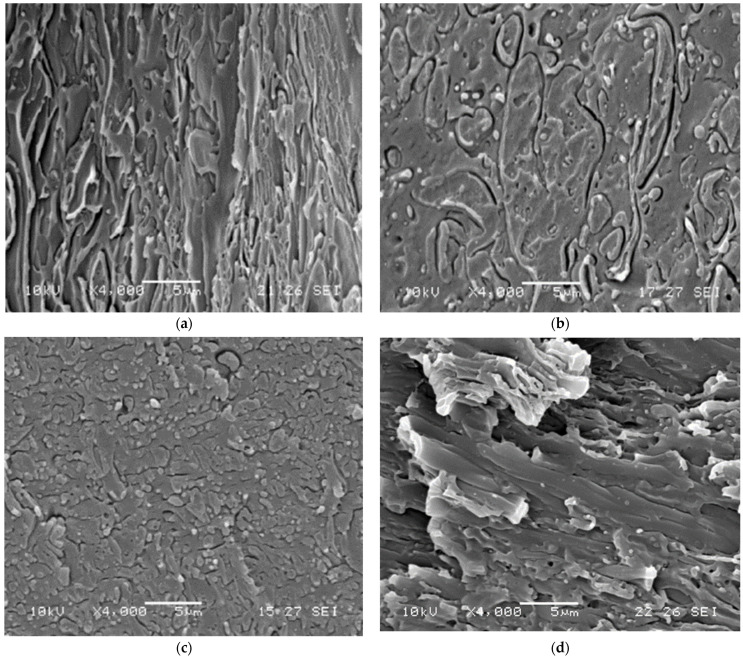
SEM Micrographs of (**a**) R-PLA60/R-PC40, (**b**) R-PLA40/R-PC60, (**c**) R-PLA60/R-PC40 + CATA, (**d**) R-PLA40/R-PC60 + CATA.

The fluidity of the polymeric melts, assessed indirectly by means of the torque measurement, necessarily goes to influence the morphological structures of these blends, which, in turn, influence the mechanical properties that are closely related. The micrographs presented in Figure 6a–d are explanatory: it can be seen in the 4000× magnifications, that the R-PLA60/R-PC40 blend (Figure 6a) is characterized by a co-continuity of phases, while the corresponding blend with the addition of TA and TBATPB (Figure 6c) shows rarefied areas of bi-continuity, but especially areas where the presence of the deformed ellipsoidal particles of PC, as dispersed phase in the PLA, is observed.

In contrast, in the case of the R-PLA40/R-PC60 blend, a PC matrix structure with spheroidal inclusions of PLA, is seen (Figure 6b), nevertheless the addition of the catalytic system caused the formation of a bi-continuous morphological structure (Figure 6d).

In the literature, it is well known that immiscible polymers, such as the PLA/PC systems, are characterized by heterogeneous morphologies achievable during the melting. The types and dimensions of the morphology determine the properties of the blend, depending on the interfacial tension, viscosities and compatibilizers [45,46]. The co-continuous structures can be considered as the coexistence of at least two adjacent structures within the same volume. The mixtures with a co-continuous structure can favorably combine the properties of both components [47] and the concept of the phase inversion must be taken into account.

Phase inversion is a phenomenon that occurs when within a mixture, as the composition changes, the polymer that had the continuous phase changes to a dispersed phase, and vice versa [48], but why is such a morphology obtained at different percentages of PLA with and without a catalyst? According to Avgeroupolos et al. [49], phase inversion occurs when the ratios of the torques and volume fractions of the components of a blend are equal. For blends without CATA, in this paper, therefore, the phase inversion point is reached for larger quantities of PLA, than for the compatibilized blends. The motivation we propose is that the lowering of the viscosity generated by the catalyst flattening the torque values to similar values, identifies the phase inversion around 50/50 between R-PLA and R-PC, for such systems causing the different behaviors, in response to the tensile stress.

**Figure 7:**
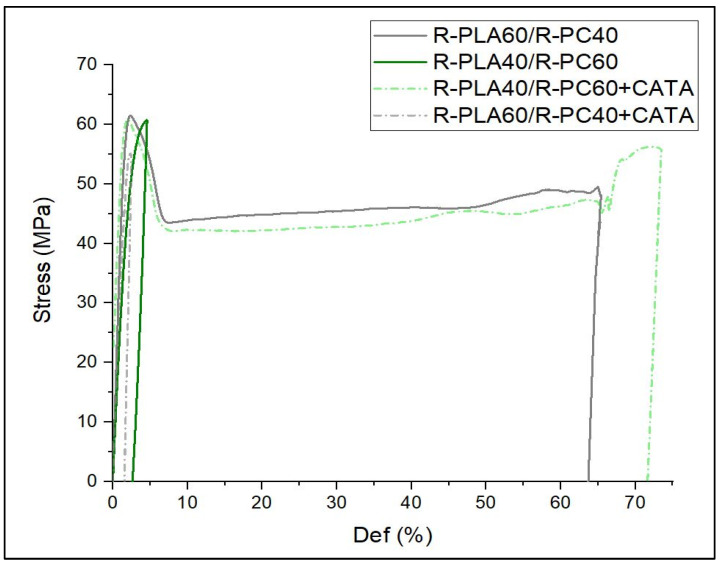
Stress-curves strain curves for the R-PLA/R-PC blends with and without CATA.

In this context Veenstra et al. [50] stated that the co-continuous morphology improves the characteristics of both polymer components, with respect to all possible morphologies. This assumption is confirmed by the quasi-static tensile properties (Figure 7 and Table 3) in which the bi-continuous blends (R-PLA60/R-PC40 and R-PLA40/R-PC60 + CATA) exhibit a much higher ductility, with elongations at break, even exceeding those of the pure PC, without decreasing in tensile strength. In contrast, the other two blends show a comparable ultimate tensile strength but with an evident brittleness. What drives the achievement of the improved properties over the pure PLA is the accomplishment of a co-continuous morphology during the processing.

The elastic modulus exhibits higher values for the mixtures with CATA; this is due to the formation of bonds, due to the interchange reaction between the components. In a previous study [22], it was seen that, after the quasi-static tests, the elongations at break, even greater than 120%, were achieved, but always with polycarbonate amounts of at least 60%; in the present work, with the recycled polymers, it is possible to state that this range of bi-continuity is much wider, allowing even blends with 60% PLA to have a co-continuous morphology, resulting in its ductile behavior.

**Figure 8:**
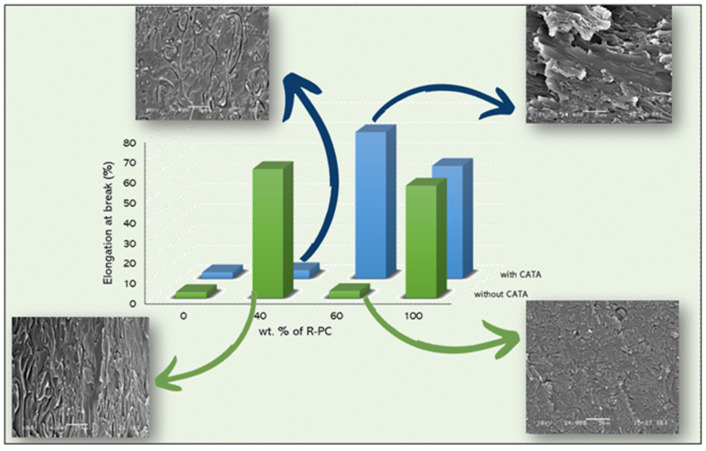
Morphology/elongation at break relationship.

This strong relationship between the phase morphology and the elongation at break values for the studied blends, has been highlighted in Figure 8.

**Figure 9:**
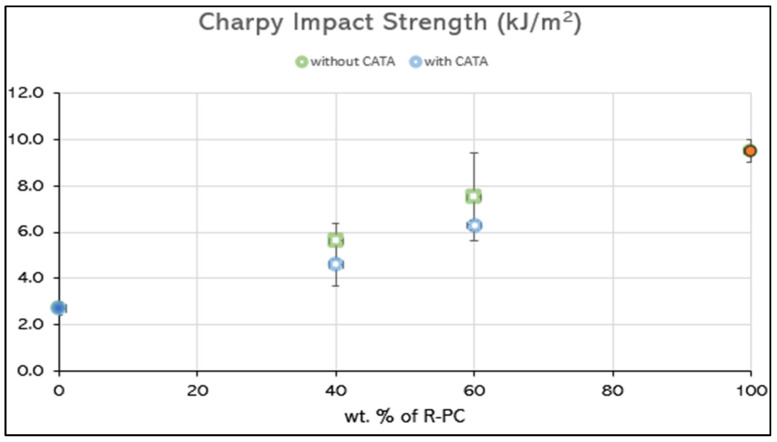
Trend of the Charpy impact strength over the R-PC quantity in the blend.

Conversely, when these materials were examined at high-speeds, through the impact tests, it has been noticed that what increases the toughness is the amount of R-PC in the compound, rather than the morphology; in fact, the trend is almost linear for both blends without and with a catalyst (Figure 9). This difference, in response to the slow test, versus the fast test, has also been found in other polymer systems, such as PLA/PBAT [51] or PLA/POE-g-GMA [52].

The catalytic system, operating through the chain rupture, did not favor the achievement of the impact strengths equivalent to the blends without a catalyst.

Definitely, as a result of the mechanical response in the slow test, specifically the tensile toughness is increased when a bi-continuous structure has been achieved during the processing, while in the fast test (impact test) it is the higher PC content that causes high value of energy absorbed before the crack propagation.

#### 3.2.3. DMTA Analysis

The development of such blends, based on polylactic acid and polycarbonate, could have a higher impact if the studied catalytic system could form copolymers during the reactive extrusion. Even polymers suffered in the industrial recycling process. In this regard, the DMTA analysis guarantees the possibility, through the study of tan delta peaks generated by a tensile test carried out at a certain frequency and in temperature sweeps, to evaluate the formation of copolymers, as demonstrated by Liu et al. [18]

**Figure 10:**
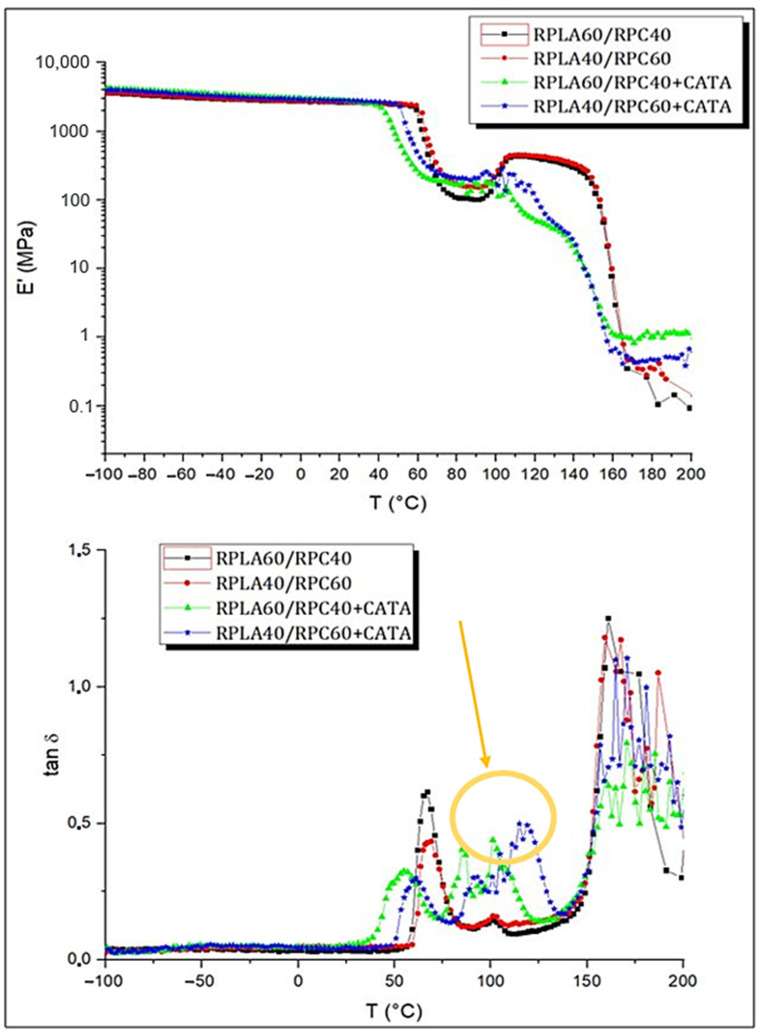
DMTA analysis to evaluate the presence of copolymers.

As shown in Figure 10, the energy released by the viscous motion of the polymer chains is reflected in the relaxation peak of tan δ, whose maximum can be considered an expression of Tg. Since the immiscible blends are those without CATA, a clear phase separation structure occurs, as revealed by two maxima in the tan δ curve. The small intermediate peaks of the blends without catalyst (red and black dots in Figure 10) are explained as the occurrence of the crystallization of the material; moreover, the more evident and significant ones (circled in yellow in Figure 10) that are present in the curves, concerning the blends with a catalyst are attributable to the formation of a copolymer that has, as Tg, a temperature intermediate between those of PLA and PC. This finding also confirms the data obtained with the virgin polymers, by Phuong et al. [22], namely that the presence of a new species is represented by the tan δ peak at around 110 °C.

Moreover, it can be noticed that the blends with the catalyst system exhibit a glass transition that starts at lower temperatures; this is probably due to the viscosity decrease caused by TA. The modulus storage E’ is higher for the mixtures with CATA, when high temperatures are reached; this is due to the bond formation because of the interchange reaction between the components.

## 4. Conclusions

The development of materials that are environmentally friendly and recyclable, that also have good mechanical properties (comparable to benchmarks), compatible with affordable price and capable to replace petroleum derivatives, is the path currently pursued in polymer research.

In this paper, the melt viscosity and mechanical properties of the virgin PLA and PC were first compared with the corresponding materials that had undergone extrusion, thermoforming processes and were recovered as production scraps. Such processing was seen to decrease the properties, but always in an acceptable range of values that guaranteed their reuse and subsequent compounding.

For this purpose, the blends with 40 and 60% wt. of recycled PLA (R-PLA) were processed by studying whether a system of compatibilizers (successfully tested on the virgin polymers in a previous paper) would also work for the blends obtained from the recycled polymers. An interesting phenomenon was seen, as a result of the mechanical response in the slow test, specifically, the tensile toughness is increased when a bi-continuous structure has been achieved during the processing, while in the fast test (impact test), it is the higher PC content that causes the high value of energy absorbed before the crack propagation. The catalytic system, through the DMTA analysis, was seen to be able to induce the formation of the PLA-PC copolymers, since the presence of a peak of the tan delta at an intermediate temperature, with respect to PLA and PC, an α-transition has been registered.

The R-PLA/RPC blends with an improved ductility, with respect to the pure recycled PLA, were obtained in the present work, thanks to the achieved R-PC phase continuity, thus suggesting the methodologies to foster the use of recycled renewable polymers in a wider range of durable applications, such as in automotive and electronic equipment, where recyclability is requested.

With respect to the blends produced by using the virgin PLA and PC [22] where 60 wt.% of PC was necessary to observe blends with a good ductility, the use of recycled polymers allowed to obtain blends with an improved ductility, using only 40% of R-PC, thus allowing to increase the renewable content (and thus the carbon storage potential) of the developed secondary material). Such compounds, therefore, represent a great opportunity because they combine a good technical potential, a high renewable content, eco-sustainability, recyclability and can be a viable solution to post-consumer disposal problems, which are increasingly burdensome in both economic and environmental terms. The challenge that needs to be addressed concerns logistics, i.e., ensuring the suitable recycling lines for materials of this type and the widespread possibility of implementing the circular-by-design concept.

## Figures and Tables

**Table 1:** Blend compositions.

Blend Name	PLA (wt. %)	PC (wt. %)	R-PLA (wt. %)	R-PC (wt. %)	TA (wt. %)	TBATPB (wt. %)
PLA	100	-	-	-	-	-
PC	-	100	-	-	-	-
R-PLA	-	-	100	-	-	-
R-PC	-	-	-	100	-	-
R-PLA60/R-PC40	-	-	60	40	-	-
R-PLA60/R-PC40 + CATA	-	-	56.8	38	5	0.2
R-PLA40/R-PC60	-	-	40	60	-	-
R-PLA40/R-PC60 + CATA	-	-	38	56.8	5	0.2

**Table 2:** Resumé of the mechanical properties for virgin and recycled polymers.

Polymer	Elastic Modulus (GPa)	Yield Stress (MPa)	Stress at Break (MPa)	Elongation at Break (%)	Charpy Impact Strength (kJ/m^2^)
PLA	3.5 ± 0.3	-	67.3 ± 3.5	2.5 ± 0.4	2.9 ± 0.2
R-PLA	3.6 ± 0.2	-	65.1 ± 3.2	3.0 ± 0.6	2.7 ± 0.3
PC	2.2 ± 0.1	67.1 ± 0.8	63.1 ± 3.8	60.6 ± 3.2	10.9 ± 0.2
R-PC	2.3 ± 0.2	65.7 ± 1.6	54.8 ± 3.9	55.7 ± 4.5	9.4 ± 0.5

**Table 3:** Resumé of the mechanical properties for the blends with the recycled polymers.

Blend	Elastic Modulus (GPa)	Yield Stress (MPa)	Stress at Break (MPa)	Elongation at Break (%)	Charpy Impact Strength (kJ/m^2^)
R-PLA60/R-PC40	2.7 ± 0.4	61.1 ± 1.1	57.2 ± 2.0	63.9 ± 8.7	7.5 ± 1.9
R-PLA60/R-PC40 + CATA	3.0 ± 0.2	-	60.1 ± 0.7	4.0 ± 0.6	6.3 ± 0.2
R-PLA40/R-PC60	3.2 ± 0.3	-	63.6 ± 2.8	3.5 ± 0.2	5.6 ± 0.8
R-PLA40/R-PC60 + CATA	3.4 ± 0.3	61.7 ± 1.5	66.4 ± 3.5	72.4 ± 9.5	4.6 ± 0.9

## Data Availability

Not applicable.

## References

[1] Muñoz Meneses R.A., Cabrera-Papamija G., Machuca-Martínez F., Rodríguez L.A., Diosa J.E., Mosquera-Vargas E. (2022). Plastic recycling and their use as raw material for the synthesis of carbonaceous materials. Heliyon.

[2] Mazur-Wierzbicka E. (2021). Circular economy: Advancement of European Union countries. Environ. Sci. Eur..

[3] Kumar R., Verma A., Shome A., Sinha R., Sinha S., Jha P.K., Kumar R., Kumar P., Shubham, Das S. (2021). Impacts of Plastic Pollution on Ecosystem Services, Sustainable Development Goals, and Need to Focus on Circular Economy and Policy Interventions. Sustainability.

[4] Farah S., Anderson D.G., Langer R. (2016). Physical and mechanical properties of PLA, and their functions in widespread applications—A comprehensive review. Adv. Drug Deliv. Rev..

[5] Naser A.Z., Deiab I., Defersha F., Yang S. (2021). Expanding Poly(lactic acid) (PLA) and Polyhydroxyalkanoates (PHAs) Applications: A Review on Modifications and Effects. Polymers.

[6] Yang Y., Zhang L., Xiong Z., Tang Z., Zhang R., Zhu J. (2016). Research progress in the heat resistance, toughening and filling modification of PLA. Sci. China Chem..

[7] Zhao X., Hu H., Wang X., Yu X., Zhou W., Peng S. (2020). Super tough poly(lactic acid) blends: A comprehensive review. RSC Adv..

[8] Liu H., Zhang J. (2011). Research progress in toughening modification of poly(lactic acid). J. Polym. Sci. Part B Polym. Phys..

[9] Lin L., Deng C., Wang Y. (2015). Improving the impact property and heat-resistance of PLA/PC blends through coupling molecular chains at the interface. Polym. Adv. Technol..

[10] Yuryev Y., Mohanty A.K., Misra M. (2017). Novel biocomposites from biobased PC/PLA blend matrix system for durable applications. Compos. Part B Eng..

[11] Nagarajan V., Mohanty A.K., Misra M. (2016). Perspective on Polylactic Acid (PLA) based Sustainable Materials for Durable Applications: Focus on Toughness and Heat Resistance. ACS Sustain. Chem. Eng..

[12] Zeng J.-B., Li K.-A., Du A.-K. (2015). Compatibilization strategies in poly(lactic acid)-based blends. RSC Adv..

[13] Srithep Y., Rungseesantivanon W., Hararak B., Suchiva K. (2014). Processing and characterization of poly(lactic acid) blended with polycarbonate and chain extender. J. Polym. Eng..

[14] Wang Y., Chiao S.M., Lai M.-T., Yang S.-Y. (2013). The role of polycarbonate molecular weight in the poly(L-lactide) blends compatibilized with poly(butylene succinate-co-L-lactate). Polym. Eng. Sci..

[15] Chen Y., Zeng G.S., Jiang P., Lu W., Huang W.L. (2011). Study on the Thermal and Rheological Properties of Reactive Blending PC/PLA/EVA Blends. Appl. Mech. Mater..

[16] Ikehara T., Nishikawa Y., Nishi T. (2003). Evidence for the formation of interpenetrated spherulites in poly(butylene succinate-co-butylene carbonate)/poly(l-lactic acid) blends investigated by atomic force microscopy. Polymer.

[17] Wang Y., Chiao S.M., Hung T.-F., Yang S.-Y. (2012). Improvement in toughness and heat resistance of poly(lactic acid)/polycarbonate blend through twin-screw blending: Influence of compatibilizer type. J. Appl. Polym. Sci..

[18] Liu C., Lin S., Zhou C., Yu W. (2013). Influence of catalyst on transesterification between poly(lactic acid) and polycarbonate under flow field. Polymer.

[19] Tripathi N., Misra M., Mohanty A.K. (2021). Durable Polylactic Acid (PLA)-Based Sustainable Engineered Blends and Biocomposites: Recent Developments, Challenges, and Opportunities. ACS Eng. Au.

[20] Pompe G., Häußler L. (1997). Investigations of transesterification in PC/PBT melt blends and the proof of immiscibility of PC and PBT at completely suppressed transesterification. J. Polym. Sci. Part B Polym. Phys..

[21] Ignatov V.N., Carraro C., Tartari V., Pippa R., Scapin M., Pilati F., Berti C., Toselli M., Fiorini M. (1997). PET/PC blends and copolymers by one-step extrusion: 2. Influence of the initial polymer composition and type of catalyst. Polymer.

[22] Phuong V.T., Coltelli M.-B.B., Cinelli P., Cifelli M., Verstichel S., Lazzeri A. (2014). Compatibilization and property enhancement of poly(lactic acid)/polycarbonate blends through triacetin-mediated interchange reactions in the melt. Polymer.

[23] Phuong V.T., Gigante V., Aliotta L., Coltelli M.B., Cinelli P., Lazzeri A. (2017). Reactively extruded ecocomposites based on poly(lactic acid)/bisphenol A polycarbonate blends reinforced with regenerated cellulose microfibers. Compos. Sci. Technol..

[24] Aryan V., Maga D., Majgaonkar P., Hanich R. (2021). Valorisation of polylactic acid (PLA) waste: A comparative life cycle assessment of various solvent-based chemical recycling technologies. Resour. Conserv. Recycl..

[25] Yarahmadi N., Jakubowicz I., Enebro J. (2016). Polylactic acid and its blends with petroleum-based resins: Effects of reprocessing and recycling on properties. J. Appl. Polym. Sci..

[26] Freeland B., McCarthy E., Balakrishnan R., Fahy S., Boland A., Rochfort K.D., Dabros M., Marti R., Kelleher S.M., Gaughran J. (2022). A Review of Polylactic Acid as a Replacement Material for Single-Use Laboratory Components. Materials.

[27] Penco M., Lazzeri A., Phuong V.T., Cinelli P. (2014). Copolymers Based on Polyester and Aromatic Polycarbonate. U.S. Patent.

[28] Tejada-Oliveros R., Gomez-Caturla J., Sanchez-Nacher L., Montanes N., Quiles-Carrillo L. (2021). Improved Toughness of Polylactide by Binary Blends with Polycarbonate with Glycidyl and Maleic Anhydride-Based Compatibilizers. Macromol. Mater. Eng..

[29] Guessoum M., Chelghoum N., Haddaoui N. (2019). Reactive Compatibilization of Poly(lactic acid) and Polycarbonate Blends Through Catalytic Transesterification Reactions. Int. J. Comput. Exp. Sci. Eng..

[30] You W., Yu W. (2018). Control of the dispersed-to-continuous transition in polymer blends by viscoelastic asymmetry. Polymer.

[31] Santi C.R., Hage E., Correa C.A., Vlachopoulos J. (2009). Torque viscometry of molten polymers and composites. Appl. Rheol..

[32] Chiu H.T., Huang J.K., Kuo M.T., Huang J.H. (2018). Characterisation of PC/ABS blend during 20 reprocessing cycles and subsequent functionality recovery by virgin additives. J. Polym. Res..

[33] Abbås K.B. (1980). Thermal degradation of bisphenol A polycarbonate. Polymer.

[34] Abbås K.B. (1981). Degradational effects on bisphenol A polycarbonate extruded at high shear stresses. Polymer.

[35] Cuadri A.A., Martín-Alfonso J.E. (2018). Thermal, thermo-oxidative and thermomechanical degradation of PLA: A comparative study based on rheological, chemical and thermal properties. Polym. Degrad. Stab..

[36] Khademi S.M.H., Hemmati F., Aroon M.A. (2020). An insight into different phenomena involved in continuous extrusion foaming of biodegradable poly(lactic acid)/expanded graphite nanocomposites. Int. J. Biol. Macromol..

[37] Polidar M., Metzsch-Zilligen E., Pfaendner R. (2022). Controlled and Accelerated Hydrolysis of Polylactide (PLA) through Pentaerythritol Phosphites with Acid Scavengers. Polymers.

[38] Signori F., Coltelli M.-B., Bronco S. (2009). Thermal degradation of poly(lactic acid) (PLA) and poly(butylene adipate-co-terephthalate) (PBAT) and their blends upon melt processing. Polym. Degrad. Stab..

[39] Coltelli M.-B., Mallegni N., Rizzo S., Cinelli P., Lazzeri A. (2019). Improved Impact Properties in Poly(lactic acid) (PLA) Blends Containing Cellulose Acetate (CA) Prepared by Reactive Extrusion. Materials.

[40] Coltelli M.-B., Toncelli C., Ciardelli F., Bronco S. (2011). Compatible blends of biorelated polyesters through catalytic transesterification in the melt. Polym. Degrad. Stab..

[41] Pérez J.M., Vilas J.L., Laza J.M., Arnáiz S., Mijangos F., Bilbao E., Rodríguez M., León L.M. (2010). Effect of reprocessing and accelerated ageing on thermal and mechanical polycarbonate properties. J. Mater. Process. Technol..

[42] Reich M.J., Woern A.L., Tanikella N.G., Pearce J.M. (2019). Mechanical Properties and Applications of Recycled Polycarbonate Particle Material Extrusion-Based Additive Manufacturing. Materials.

[43] Agüero A., Morcillo M.d.C., Quiles-Carrillo L., Balart R., Boronat T., Lascano D., Torres-Giner S., Fenollar O. (2019). Study of the Influence of the Reprocessing Cycles on the Final Properties of Polylactide Pieces Obtained by Injection Molding. Polymers.

[44] Bi F.-L., Xi Z.-H., Zhao L. (2018). Reaction Mechanisms and Kinetics of the Melt Transesterification of Bisphenol-A and Diphenyl Carbonate. Int. J. Chem. Kinet..

[45] Sadiku-Agboola O., Sadiku E.R., Adegbola A.T., Biotidara O.F. (2011). Rheological Properties of Polymers: Structure and Morphology of Molten Polymer Blends. Mater. Sci. Appl..

[46] Willemse R.C., Posthuma de Boer A., van Dam J., Gotsis A.D. (1999). Co-continuous morphologies in polymer blends: The influence of the interfacial tension. Polymer.

[47] Pötschke P., Paul D.R. (2003). Formation of Co-continuous Structures in Melt-Mixed Immiscible Polymer Blends. J. Macromol. Sci. Part C.

[48] Yeo L.Y., Matar O.K., de Ortiz E.S.P., Hewitt G.F. (2000). Phase Inversion and associated phenomena. Multiph. Sci. Technol..

[49] Avgeropoulos G.N., Weissert F.C., Biddison P.H., Böhm G.G.A. (1976). Heterogeneous Blends of Polymers. Rheology and Morphology. Rubber Chem. Technol..

[50] Veenstra H., Verkooijen P.C.J., van Lent B.J.J., van Dam J., de Boer A.P., Nijhof A.P.H.J. (2000). On the mechanical properties of co-continuous polymer blends: Experimental and modelling. Polymer.

[51] Gigante V., Canesi I., Cinelli P., Coltelli M.B., Lazzeri A. (2019). Rubber toughening of Polylactic acid (PLA) with Poly(butylene adipate-co- terephthalate) (PBAT): Mechanical properties, fracture mechanics and analysis of brittle—Ductile behavior while varying temperature and test speed. Eur. Polym. J..

[52] Aliotta L., Gigante V., Acucella O., Signori F., Lazzeri A. (2020). Thermal, mechanical and micromechanical analysis of PLA/PBAT/POE-g-GMA extruded ternary blends. Front. Mater..

